# Supplementary material for: Short-Chain Fatty Acids Attenuate 5-Fluorouracil-Induced THP-1 Cell Inflammation through Inhibiting NF-κB/NLRP3 Signaling via Glycerolphospholipid and Sphingolipid Metabolism
Source: Molecules. 2023 Jan 4;28(2):494. doi: 10.3390/molecules28020494 (PMC9864921; doi:10.3390/molecules28020494)
Supplement: Supplementary file 1 [file molecules-28-00494-s001.zip › molecules-2128710-supplementary.pdf]

Supplementary Material

Short-Chain Fatty Acids Attenuate 5-Fluorouracil-Induced THP-1 Cell Inflammation through Inhibiting NF-κB/NLRP3 Signaling via Glycerolphospholipid and Sphingolipid Metabolism

Yanyan Zhang [1,†], Yue Xi [2,†], Changshui Yang [3], Weijuan Gong [3,*], Chengyin Wang [1], Liang Wu [4] and Dongxu Wang [5,*]

[1] Testing Center, Yangzhou University, Yangzhou 225009, China
[2] Medical Laboratory Department, Huai'an Second People's Hospital, Huai'an 223022, China
[3] School of Medicine, Yangzhou University, Yangzhou 225009, China
[4] Department of Laboratory Medicine, School of Medicine, Jiangsu University, Zhenjiang 212013, China
[5] School of Grain Science and Technology, Jiangsu University of Science and Technology, Zhenjiang 212100, China
* Correspondence: wjgong@yzu.edu.cn (W.G.); wdx@just.edu.cn (D.W.)
† These authors contributed equally to this work.

Table S1. RT-PCR Primers sequence.

Gene	Primer sequence (5'→3')
GAPDH	FP: CATCACTGCCACCCAGAAGACTG
	RP: ATGCCAGTGAGCTTCCCGTTCAG
NLRP3	FP: AACAGCCACCTCACTTCCAG
	RP: CCAACCACAATCTCCGAATG
Caspase-1	FP: GCACAAGACCTCTGACAGCA
	RP: TTGGGCAGTTCTTGGTATTC
IL-1β	FP: CCTGTCCTGCGTGTTGAAAGA
	RP: GGGAACTGGGCAGACTCAAA
IL-6	FP: CCTTCGGTCCAGTTGCCTTCT
	RP: GAGGTGAGTGGCTGTCTGTGT
IL-10	FP: TCTCCGAGATGCCTTCAGCAGA
	RP: TCAGACAAGGCTTGGCAACCCA

Note: FP: forward primer; RP: reverse primer.
